# Dr. Marshall Hall on a Serious Morbid Affection, Chiefly Occurring after Delivery, Miscarriage, &c.

**Published:** 1820-09-01

**Authors:** 


*Cases of a serious morbid Affection, chiefly occurring after Delivery, Miscarriage, &c. from various Causes of Irritation and Exhaustion; and of a similar Affection, unconnected with the Puerperal State.* By Marshall Hall, M.D. F.R.S.E. &c. &c. Octavo, pp. 88. London, 1820.

The line which separates spasm and irritation from inflammation, is often so obscure, that the man who could lay down an accurate diagnosis on this subject, would do a very great service to the medical profession. We believe it is a maxim pretty generally acted upon in practice, to treat a disease as inflammation, when we are in doubt whether it be or be not spasm. This, upon the whole, is a safe rule; but cases occasionally present themselves, where it is little less destructive to bleed in irritation, than to stimulate in inflammation. It appears to be the object of Dr. Hall, whose talent for observation and power of discrimination are well known, to draw the attention of his brethren to the above-mentioned cases, by a plain record of facts. The motto which he has prefixed to his work, taken from Dr. Denman, is very appropriate, and is as follows:

"When the puerperal fever of a true inflammatory nature exists, I feel I am right in the opinion I have advanced respecting bleeding. But as it is sometimes EXTREMELY DIFFICULT TO DISTINGUISH BETWEEN THIS FEVER AND COMPLAINTS PROCEEDING FROM MERE IRRITABILITY, which far more frequently occur, especially in very delicate habits, and among women of high rank; and as all the complaints arising from irritability would at this time be INCREASED BY BLEEDING, AND RENDERED DANGEROUS BY A REPETITION OF IT, I recommend, in the strongest terms, that we should be ACCURATE IN OUR DISTINCTIONS before we determine on a plan, on our reliance and pursuit of which the good of our patient may so essentially depend."—*Denman's Midwifery.*

Upon the above passage, from Dr. Denman, we will only remark, that it is not very probable we shall be "*accurate* in our distinctions," where it is "extremely difficult to distinguish." This remark is not meant to deter from the attempt at diagnosis, but to show how easy it is to recommend on paper, what is scarcely possible in practice.

The morbid affection which forms the subject of the following work has, Dr. H. thinks, been unaccountably neglected by authors who have written on midwifery and puerperal diseases, a circumstance the more extraordinary, if the statement of Dr. Hall be correct; "namely, *that the morbid af-*

*fection in question constitutes a great proportion among puerperal cases, and a great majority among the fatal ones, and of these fatal cases, many are daily rendered so by a mistaken use of the lancet.*"

This is a very strong statement; and we are tempted to believe, that it results from experience in a peculiar locality, such as Nottingham, where sedentary habits, poverty, and the depressing passions, operate powerfully in rendering the constitution remarkably irritable, and unable to bear that vigorous system of depletion which, under other circumstances, has been found so necessary in arresting the progress of puerperal affections. We throw out this hint, not in opposition to Dr. Hall's inferences, but to put our brethren on their guard, and to induce them to study well the constitutions of their patients, and their habits of life, before they implicitly adopt our author's conclusions, however legitimately they may be drawn, under the circumstances in which he is placed.

Dr. Hall observes, that whilst this affection arises from various sources of irritation and exhaustion, it is apt to produce symptoms resembling those of increased action, prompting to evacuations which, exasperating the disease, lead to still bolder measures of depletion, and, in some instances, to the destruction of life. As the puerperal state is peculiarly liable to causes both of irritation and inflammation; and as the *symptoms* of these are very similar, while their nature and treatment are different, the subject, from this consideration, acquires great interest. Dr. Hall deprecates the idea of inculcating the neglect of blood-letting, in cases of actual inflammation—his object is to institute a rigid diagnosis—"and the greatest vigilance in regard to the effects of bleeding, should this measure be once instituted." vii.

I. *Etiology.* The principal source of *irritation*, is a disordered and loaded state of the alimentary canal; that of *exhaustion*, uterine hæmorrhagy. The disease is more particularly apt to occur in females whose bowels had been in an irregular state, whether from constipation or diarrhœa, prior to delivery. It is frequently, according to our author, induced by copious or protracted uterine hæmorrhage, menorrhagia lochialis, long-continued lactation, sickness, copious venesection for other complaints, misapplied blood-letting after confinement, and the violent operation of purgatives.

It is most prone to attack the delicate and feeble; being aggravated, or even induced by closeness and warmth of the patient's room, the fatigue of lingering labour, anxiety of mind, alarm and hurry.

II. *Symptomatology.* When *acute*, the first symptom is usually a severe and protracted rigor, succeeded by a hot stage, "and some serious affection of the head or abdomen. When the attack is slower and more insidious, the rigor is less observable, the heat of surface is absent perhaps, and there is throbbing pain of the head, with vertigo, when in the erect posture, or fluttering and palpitation of the heart, or oppressive, hurried, and sighing respiration, or irritability of the stomach and bowels."

Of the two principal causes of the disease under review, intestinal irritation and uterine hæmorrhage, the *former* more commonly produces pain of the head, side, iliac region, loins, or some part of the abdomen; the *latter*, faintishness, gasping for air, jactitation, sense of sinking, &c.

"But, besides that these two causes generally co-exist, and co-operate, it is remarkable how similar, in general, are their effects. They each seem liable to attack ALL the organs and functions of the body, conjointly or separately. It is, however, in general, the circumstance of EXHAUSTION, which adds REAL DANGER to the otherwise URGENT but LESS SERIOUS symptoms of IRRITATION," xxvii.

The symptoms which may be referred to the HEAD, are severe pain, beating and throbbing, vertigo, intolerance of light and noise, wakefulness, starting from sleep, with faintness, and sense of impending dissolution; sometimes delirium.

When the HEART is affected, its functions are, of course, disturbed, and all kinds of irregularity in the circulation, ensue.

III. *Terminology.* This disease (for which we have no other name than "puerperal affection)" sometimes terminates in sudden and unexpected dissolution.

"Is it not, (says our author) in fact, by this complaint that the nation has been bereaved of a princess on whom every hope and heart was fixed, and has been plunged into lasting mourning?"

Sometimes the affection terminates fatally, after a more or less urgent or protracted course. "Lastly, this affection has frequently yielded favourably to the resources of art;" which Dr. H. is happy to say, was almost invariably the case, when the plan of treatment, hereafter to be mentioned, was pursued.

IV. *Diagnosis.* It is most apt to be mistaken and mistreated for inflammatory diseases of the head, chest, heart, stomach, bowels, uterus, and peritoneum; but especially for puerperal fever and puerperal phrenitis. This fact, our author thinks, may tend to explain the discrepancy of opinions respecting the treatment of puerperal fever by blood-letting. For the *diagnosis*, Dr. Hall refers us to the symptomatology already detailed; but on re-perusing the sections which Dr. H. alludes to, we confess that we are unable to state what the diagnostics are. We are, therefore, fully agreed with our author in the following sentiment.

"The diagnosis may frequently be attended with difficulty; there will often be occasion for the most careful and anxious WATCHING, and for the utmost PRUDENCE in the administration of remedies, and in the observation of their effects." xxxvii.

Where blood-letting is had recourse to, the effect of the measure must be rigidly observed, and early faintness, increase of frequency in the pulse, gasping, feeling of dissolution, or unremitting pain, must render us cautious how we proceed.

"In all cases, the colon and rectum should be unloaded by enemata. This measure affords a source of diagnosis of the utmost importance, in the relief it confers, and in the opportunity it gives for the observation of the state of the intestinal canal." xl.

V. *Treatment.* This consists, very properly, in removing the causes, and obviating the effects of irritation or exhaustion. Intestinal irritation must be removed by aperient medicines and enemata. Small doses of calomel, and draughts, with rhubarb and sulphate of magnesia, are the best aperients, according to our author's experience.

"One point is of the greatest importance; it is the union with the purgative, of a proper dose of opium, or of a stimulant medicine; and a point of little inferior importance, is the administration before, during, and after the action of the purgative medicine, of proper nourishment. With the calomel, I have given a small quantity of opium, and with the rhubarb and sulphat of magnesia, a little of the tinctura cardamomi comp." xliv.

Dr. Hall finds it impossible to say too much in recommending enemata. By their means the intestine is unloaded effectually, and without the exhaustion experienced from the action of efficient purgative medicines, or the danger of disordering the stomach.

"It is, in the next place, necessary to notice the means for obviating the various sources of exhaustion. If this be uterine hæmorrhagy, the following application is, I think, most effectual in arresting it. A lotion is prepared by dissolving from one to two drachms and a half of sulphat of zinc in a pint of soft water; a scroll of linen is then made of a proper form and bulk to fill the vagina; this scroll is then fully imbued with the lotion, introduced into the vagina, and renewed frequently. The same lotion may also be applied externally." xlvi.

The other remedies which our author found useful were tinct. opii, tinct. camphoræ compos. the spir. ammoniæ aromaticæ, ether, wine, and similar remedies. A proper combination of these remedies induces quiet sleep, prevents the exhaustion which would otherwise ensue from the administration of purgatives, and relieves many distressing symptoms. Gentle unirritating nourishment must, of course, be given; as chicken broth, arrow root and milk, in small quantities, every hour, or oftener.

Such is the general treatment; but what is to be done in the case of severe local affection and pains? When the *head* is much affected, Dr. H. recommends cold lotions to that, and hot fomentations to the feet. He thinks leeches may sometimes be necessary, "when the complaint is the effect of *irritation;* but they can only palliate." When the head is affected from *exhaustion,* even leeches are hazardous, and venesection ought, he thinks, to be proscribed. When the abdomen is the seat of the disease, "enemata, purgative and opiate, fomentations of the part and of the feet, liniments, and leeches, are the remedies of greater efficacy as well as safety." Venesection, in these cases, aggravated the pain, and proved hazardous to life.

"In the attack of jactitation, of oppression of the breathing, of palpitation, of faintishness, &c. a draught with tinctura opii, sp. ammoniæ aromat. opening the door and window, fanning, bathing the temples with vinegar, the smelling bottle, and aromatic vinegar, are all important." lii.

The best preventive of these dangerous visitations, is quietude of mind and body after delivery.

The morbid affection in question may occur independently of the puerperal state, particularly after great uterine discharges, protracted lactation, &c. and where a deranged state of the stomach and bowels, with intestinal irritation, obtain.

"When this affection arises from a disordered or loaded state of the stomach or bowels, purgatives, followed, or accompanied by opiates, and enemata, are the remedies on which most reliance is to be placed.

"In the other cases, the tinctura opii, the sp. ammoniæ aromat.; wine; stimulating liniments; proper fluid nourishment, cautiously given with wine; bathing the face with cold water; the effervescing medicine; fanning, and a free air, are the principal remedies." lxxiii.

The great body of the work (62 pages) consists of a detail of cases, illustrative of the foregoing observations. Some of these we shall shorten or extract, to make this article more complete.

"*Case* 1. A woman, whose labour was lingering, was bled to eighteen ounces, which expedited the delivery. She continued well till next morning, when she was seized with repeated shiverings, succeeded by pyrexia and gastric irritability. An enema and purgative medicine brought away much hardened fæces, and a fluid resembling yolk of egg, with great relief. In the evening, however, there was heat, restlessness, jactitation, slight delirum, head-ache, confused vision, and faintness on attempting to sit up. *Effervescing mixture with salts.*—Third morning. The sickness returned with much uterine discharge. The opening medicine had acted. Thirty-five drops of tinctura opii in a draught. At eight o'clock in the morning, Dr. H. saw the patient, when she had nearly the same symptoms as described above. Thirty-five drops of tinctura opii, with a dram of the sp. ammon. aromat. administered. Cold zinc lotion to the pubis and vagina.—*Evening.* Had had some refreshing sleep; all the symptoms were abated; the bowels had acted, and the uterine discharged was lessened. She was ordered to take ten drops of the tincture of opium, and half a dram of the sp. ammon. aromat. every five hours; the effervescing medicine to be continued. After some slight affections of the chest and limbs, which were removed by anodynes, the patient recovered."

The following case will be found interesting in many points of view.

*Case* 2. Mrs. C. aged 21, was confined on a Saturday, in June, 1819, of her first child. Much scybalous fæces were passed during labour, and on the succeeding day, from ℥jß of ol. ricini. No more motions till Thursday, when she became affected with feverish heat, noise in the head, vertigo; the countenance was flushed; pulse 120, with beating of the carotids; flashings of light before the eyes; respiration rather quick; lochia and milk abated; tongue loaded. No pain. Fourteen ounces of blood from the arm induced deliquium. She felt relieved, and the pulse fell. A cold lotion was applied to the forehead, a blister to the neck, and a powerful purgative given.

About seven hours afterwards, the pulse got up to 120, with noise in the head; the pupils continuing natural. Twelve ounces of blood abstracted while in the erect posture, which produced fainting. Five grains of calomel were given, and an enema with an ounce of ol. terebinthinæ. Copious dark and very offensive motions were passed in the night. At seven o'clock on Friday morning, the pulse was down to 100; the head relieved; the skin cool; the tongue white and moist. The opening medicine was continued.

On Saturday, there being some head-ache, with slight flushing of the cheeks, eight ounces of blood were taken away, which, like the preceeding, exhibited a buffy coat. Leeches and a blister were ordered; and four grains of calomel and pulv. antim. were given every four hours.

On Sunday morning the practitioner found her with violent beating in the head; pulse 120; the skin very hot; a great expression of lowness in the countenance; lochia and milk stopped. A tea-cupful of blood taken produced faintness, and abated the beating in the head. Six drops of the black drop given. About noon she was flushed, and the beating had increased. Six ounces of blood were drawn from the nape of the neck by cupping. The calomel and antimony continued. Prussic acid prescribed. — *Monday,* the pulse stronger, but 130; sinapisms to the feet.—*Tuesday,* her head felt heavy like a brick, and she felt as though she were dying. A dose of rhubarb with sal. polychrest, followed by seven drops of the black drop. Refreshing sleep resulted. An enema exhibited, on awaking, brought away several fœtid evacuations. In the evening, felt better, another opiate administered.—On *Wednesday,* she had had several hours refreshing sleep, awoke in a profuse perspiration, and said she was quite well. Alternate doses of opiate and aperient medicines were continued for some time, and the patient recovered.

Our limits will not permit us to notice any more of the cases terminating favourably, of which there are eighteen detailed.

The following case is introduced by Dr. Hall, under the head of "fatal effects of blood-letting." We shall give it entire.

"Mrs. V. of pale and sallow complexion and weakly constitution. Six days before her confinement of her first child, she was awoke in the night by severe pain of the head confined to one spot. This pain continued several hours, when Mrs. V. applied to her medical man; she was completely relieved by losing sixteen ounces of blood, followed by purgative medicine, and she continued well.

"Mrs. V's labour occurred on September the 1st. 1817, and was rather tedious, but natural, and she had no complaint until the second day, when she experienced a second attack of pain in the head, but less violent than the previous one. She was seen six hours after this attack; she then complained of pain and beating of the head, about the anterior part of the right parietal bone: the skin was hot, and the pulse frequent and strong. Sixteen ounces of blood were taken from the arm, leeches ordered to be applied to the temples, and an enema and purgative medicine were prescribed.

"In three hours time Mrs. V. was again visited, and it was deemed necessary to abstract more blood. Six or eight ounces were therefore taken; faintishness was induced, and the symptoms were little abated.

"On the succeeding morning, September the 4th, the symptoms still remained the same; the surface was hot; the bowels had been purged, and the evacuations were natural.—The saline mixture was ordered.—At noon the symptoms remaining as before, the purgative medicine was repeated and a blister was applied.—In the evening, the evacuation of the bowels was satisfactory; the pain of the head was not severe, but there were much beating and a rushing noise; there was restlessness; and a teazing, irritative cough.—A draught with thirty drops of the tinctura opii was administered.

"The next morning, September the 5th. Mrs. V. expressed herself as being much better from having enjoyed comfortable sleep. The surface was still hot, and the head still affected as before.—In the evening, there was a degree of tenderness in the region of the uterus; she dreaded the idea of being bled, from the faintishness she had before experienced from it, and said it would certainly kill her.

"On the morning of the 6th. the pain in the region of the uterus was relieved, the head was affected as before, the window was kept open for want of air.—In the evening Mrs. V. complained of being fainty and low.—A mixture with camphor and sulphuric æther was prescribed.

"On the 7th. the irritative cough again occurred; the pulse was frequent, from 120 to 130; and the other symptoms remained unabated.—A physician was consulted.—Sixteen ounces of blood were directed to be taken from the arm; a grain of calomel was given every three hours, and the effervescing medicine was ordered.

"On the morning of the 8th. Mrs. V. appeared to be relieved in every respect; the heat of surface and the pain of the head were diminished; the blood presented the buffy coat.—It was thought proper to abstract more blood, as the last bleeding had apparently conferred benefit, and had been borne better than the preceding ones. Four tea-cupfuls of blood were taken;—the most dreadful fainting followed, with gasping, open mouth, a convulsive action of the diaphragm,—and in an hour or two death closed the scene." P. 51.

We grant, that in the foregoing case, the *last* venesection was exceedingly ill-judged, and we much wonder how any physician, of common observation and reasoning powers, could have persisted in such ultra-depletory measures, seeing that the symptoms were all relieved by the previous depletion, which previous depletion, we consider to have been not only justifiable, but indispensibly necessary. It is hardly fair then to class this case under the head of "fatal effects of blood-letting," since death was owing to an injudicious *perseverance* in the measure, rather than to any impropriety in the principle itself; for we believe, that the patient would have died, had she not been bled at all. Indeed, we have frequent occasion to deplore the ultra-depletory mania which has seized some weak brains of the present day, and much fear it will bring a character of rashness and danger on a most important remedy, when judiciously managed. This has already been the case with mercury; and, therefore, we consider it our duty to repress, as far as in our power, all extremes, whichsoever way they bend.

We shall conclude our extracts with the following case, illustrative of the affection under consideration, as occurring independently of the puerperal state.

"Mrs. Y. aged 26, had been alarmingly indisposed during fourteen days, when my attendance was requested. Straw had been spread over the pavement, and the candles were screened; she had awoke from her sleeps in great alarm, with palpitation and fainting; and, on a subsequent occasion, the windows and doors were requested to be thrown open, and the smelling bottle was used.

"Mrs. Y. was indisposed six weeks previously to the present attack. It was thought right to take away some blood. Mrs. Y. did not completely recover, but lost her good looks and continued to feel poorly. The complexion had become pallid; the bowels were constipated and disordered; the catamenia had long been profuse.

"Mrs. Y. became indisposed when at a distance from home. At 11 o'clock at night she was seized with severe suffering, begged the window might be opened, and was relieved by taking a little brandy and water. On the ensuing day Mrs. Y. was low and ill, but free from palpitation or fainting. During the next three days Mrs. Y. continued better, and on the last, performed a journey home without any bad effect.

"About 11 o'clock at night, a few days after her return, another attack was experienced, attended by palpitation, fainting, and coldness. A little brandy and water again afforded some relief.

"On the next day, eight ounces of blood were taken from the arm. Mrs. Y. continued low and ill, but free from any attack of the kind formerly experienced. On the next day the same report was made.

"At seven in the evening of the succeeding day, there was another attack, and a more formidable one at eight. On the next day there was pain of the head; and on the next, early in the morning, there was an attack of urgent suffering, feeling of dissolution, sense of heat about the head and stomach; this was relieved, but Mrs. Y. continued weak and low. A similar paroxysm took place the ensuing day, and again on the next, leaving her exhausted, restless, and wakeful. The surface was affected, with great perspiration and sensibility to the cold.

"On the succeeding day there were frequent attacks of palpitation and fainting,—especially on falling asleep.

"The following were the principal symptoms observed in this case; great susceptibility to fatigue, noise, or disturbance; sense of heat about the head and vertigo; wakefulness; palpitation and faintishness, especially on falling asleep and on awaking; great oppression in the breathing, with eructation; painful spasms, and sense of numbness or torpor of the limbs. These symptoms recurred in alarming paroxysms, and from the slightest causes.

"This affection yielded in the most favourable manner, almost without any reverse, to a cautious conjunction of aperients, opiates, the sp. ammoniæ aromat. and the tinctura camphoræ comp. and nourishment frequently given in small quantity,—every source of exertion, of hurry, of thinking, or of feeling, being vigilantly avoided." P. 57.

We have now exhibited, we hope, such an analysis of the little work before us, as will enable the reader to judge for himself respecting its merits. We have very little comment to make ourselves. We think the profession is under great obligations to Dr. Hall, for drawing their attention to those puerperal affections where irritation borders on, or even assumes the character of inflammation, and where strong depletory measures should be cautiously put in force. The only draw-back on the utility of the publication, is the danger of its embarrassing the inexperienced practitioner, where actual inflammation obtains, combined with, or under the guise of irritation. At all events, we trust that the work will tend to effect the object designed by the author, that of exciting the minutest attention to the *discrimination* of diseases.

